# Ethnic differences in realising desires to leave urban neighbourhoods

**DOI:** 10.1007/s10901-016-9524-3

**Published:** 2016-08-09

**Authors:** Sanne Boschman, Reinout Kleinhans, Maarten van Ham

**Affiliations:** 1 Department of Sociology/ICS, Utrecht University, Utrecht, The Netherlands; 2 Department OTB - Research for the Built Environment, Faculty of Architecture and the Built Environment, Delft University of Technology, Delft, The Netherlands; 3 School of Geography and Geosciences, University of St Andrews, St Andrews, UK

**Keywords:** Ethnic minorities, Moving desires, Neighbourhoods, Segregation, Selective mobility

## Abstract

Selective mobility into and out of urban neighbourhoods is one of the main driving forces of segregation. Earlier research has found group differences in who wants to leave or who leaves certain types of neighbourhoods. A factor that has received little attention so far is that some residents will have a desire to leave their neighbourhood, but are unable to do so. If there are differences between population groups in the realisation of desires to leave the neighbourhood, this might lead to involuntary segregation. This paper uses a unique combination of register data and survey data. We combine data from a large housing survey in the Netherlands (WoON) with longitudinal register data from the Netherlands (SSD) which contains individual-level information on residential mobility histories. This allows us to study whether households with a desire to leave their neighbourhood do realise this desire and which households are successful in leaving which neighbourhoods. A more thorough insight in who wants to leave which neighbourhoods but is unable to do so will contribute to a better understanding of selective mobility and segregation. We find that ethnic minorities and low-income households are less likely to realise a desire to leave their neighbourhood. We expected that ethnic minorities would be especially unsuccessful in realising desires to leave minority concentration neighbourhoods; however, for none of the ethnic groups we found an effect of neighbourhood ethnic composition on the realisation of desires to leave.

## Introduction

Selective mobility into and out of urban neighbourhoods is one of the driving forces of ethnic and socio-economic segregation. The segregation literature gives insight in the interrelatedness of neighbourhood characteristics and residential mobility. Selective residential mobility will affect neighbourhood characteristics, and in turn, neighbourhood characteristics can be a trigger to move. As Logan and Alba (1993) state, it is important to study (the causes of) ethnic or racial differences in residential outcomes, because of the strong effects the residential neighbourhood can have on social opportunities (Friedrichs et al. 2003; Wilson 1987).

Much research has focussed on features of residents who want to leave certain types of neighbourhoods or of residents actually leaving these neighbourhoods. These studies give insight in which neighbourhood characteristics are a reason to (want to) leave and how this differs between population groups. People who are different from the majority population of the neighbourhood are found to be more likely to (want to) leave the neighbourhood (Kearns and Parkes 2003; Lee et al. 1994; Musterd et al. 2014; Schaake et al. 2010; South and Crowder 1998; Van Ham and Clark 2009; Van Ham and Feijten 2008), which may result in reproduction of segregation.

However, earlier research does not tell us whether people with a desire to leave their neighbourhood are successful in realising this desire and which people manage to leave which neighbourhoods. Not only the desire to leave the neighbourhood may be selective, but also the probability of success. If there are differences between ethnic or racial groups in the wish to leave certain neighbourhoods, segregation might be voluntary. However, if individuals from one ethnic group are equally likely to want to leave, but less successful than others in leaving, this may indicate that segregation is involuntary. Segregation literature has focused on group differences in who wants to leave, but has devoted little attention to group differences in who realises these desires.

In the *residential mobility* literature, several studies analyse the relationship between moving wishes and moving behaviour. These studies reveal a large discrepancy between a desire to move and actual moving behaviour. The majority of people with a desire to move do not move within 1 or 2 years (Crowder 2001; De Groot et al. 2011; Kan 1999; Lu 1999). In Europe, ethnic minority groups are found to be especially unsuccessful in realising their desires to move (Boschman and De Groot 2011), and in the USA, Blacks are found to be less successful than Whites (Crowder 2001; Kan 1999).

In this paper, we create a link between the segregation literature and the residential mobility literature. Segregation literature has studied selectivity in who wants to leave the neighbourhood (Van Ham and Feijten 2008) or who leaves the neighbourhood (Van Ham and Clark 2009). Residential mobility literature has studied who realises desires to move. This paper focuses on people who want to leave their neighbourhood and studies selectivity in who realises their desire to leave. Thereby, we especially focus on differences between ethnic groups.

Firstly, we analyse who is successful in realising their desire to leave. In earlier research, ethnic minorities have been found to be less successful in realising moving wishes. Does this also imply that they are less successful in realising a wish to leave the neighbourhood?

Secondly, we study who are successful in *leaving which neighbourhoods*. Ethnic minorities (in Europe) as well as Blacks, Hispanics and Asians (in the American literature), have been found to be less likely than the native majority to leave poverty neighbourhoods (Bolt and Van Kempen 2003; Quillian 2003; South et al. 2005) or minority concentration neighbourhoods (Bolt and Van Kempen 2010; Pais et al. 2009). An important question is whether ethnic minorities are less successful than others in leaving these neighbourhoods, also *if they have expressed a desire to leave.* Both the residential mobility and segregation literatures will benefit from more insights in the characteristics of people who are (un)able to leave undesired neighbourhoods.

This paper uses a combination of register data and survey data. We use data from a large housing survey in the Netherlands (WoON) on the wish to leave the neighbourhood, and we combine these data with longitudinal register data from the Netherlands (SSD), which contains individual-level information on residential mobility histories. This combination of data sets allows us to study which households with a desire to leave their neighbourhood subsequently realise their desire.

In the next section, we discuss the theory on urban segregation and residential mobility, creating a link between these two fields of study, and formulate hypotheses about expected group differences in success. Section three describes the used register and survey data and the methods. In section four, we present logistic regression models explaining the realisation of desires to leave the neighbourhood from personal characteristics, neighbourhood characteristics and interactions. The last section draws conclusions on group differences in realisation of desires to leave and their effect on segregation.

## Theory

Segregation refers to the unequal distribution of population groups over space. Selective residential mobility is one of the driving forces of segregation. Starting with the Chicago School (Park et al. 1925), many researchers have described the nature of urban segregation and the role of selective mobility patterns in (re)producing segregation (Clark 1991; Hedman et al. 2011; Sampson 2012; Schelling 1971; Van Ham et al. 2012). To understand selective mobility patterns, researchers have tried to gain insight in individual differences in mobility behaviour.

Many researchers have found ethnic or racial differences in residential mobility behaviour and outcomes. Blacks are found to be less likely than Whites to move to suburbs (Logan and Alba 1993) and more likely to move to poverty neighbourhoods (Clark et al. 2006) or Black concentration neighbourhoods (Clark and Ledwith 2007; South and Crowder 1998). Also in Europe, ethnic minorities are found to be more likely than natives to move to poverty neighbourhoods (Bolt and Van Kempen 2003; Schaake et al. 2014) or minority concentration neighbourhoods (Bråmå 2006; Doff 2010). Similarly, ethnic minority groups, or Blacks, Hispanics and Asians, are found to be less likely to *leave* ethnic minority concentration neighbourhoods (Bolt and Van Kempen 2010; Pais et al. 2009; South and Crowder 1998) or poverty neighbourhoods (Bolt and Van Kempen 2003; Quillian 2003; South and Crowder 1997; South et al. 2005). Ethnic or racial differences in mobility behaviour can be partly explained by socio-economic differences between groups. Due to their lower income, ethnic minorities more often move to neighbourhoods with low dwelling values or high shares of social rented dwellings, which are also more often minority concentration neighbourhoods (Boschman and Van Ham 2015). However, also when socio-economic differences are taken into account, ethnic minorities are found to be less likely than majority group members to move to high-income or majority concentration neighbourhoods (Schaake et al. 2014). To understand individual mobility behaviour and the relation between moving desires and their realisation, insight is needed in the residential mobility literature.

### Residential mobility

Researchers from Rossi (1955) onwards have attempted to describe and explain individual residential mobility processes. Early theorists assumed that a discrepancy between the preferred and the actual housing situation leads to residential stress or dissatisfaction (Speare et al. 1974; Wolpert 1965), and if residential stress reaches a threshold level, it will trigger a desire to move (Brown and Moore 1970). Households with a desire to move will search for housing opportunities that better fulfil their residential needs (Brown and Moore 1970). However, moving desires will not always be fulfilled. Some groups will be more successful than others in realising their desire to move (Lu 1999). Many factors compound the relation between satisfaction, moving intentions and actual moves, and thus result in behavioural inconsistencies in residential mobility (Coulter et al. 2012; De Groot et al. 2011; Lu 1999). Whether households will be able to translate mobility desires into an actual move depends on their personal preferences, resources and restrictions, as well as the opportunities and limitations imposed by the local housing market (Mulder and Hooimeijer 1999).

A high income increases the opportunities to improve the housing situation, while renters can more easily move because their transaction costs related to the move are much lower than for owner-occupiers (Mulder and Hooimeijer 1999; Murie 1974; Priemus 1984). Larger households have higher moving costs and have to take into account accessibility of jobs, schools and facilities for all household members when searching a new dwelling (Schwartz 1973). Large households will thus be less successful in realising their moving wishes, also because they are more constrained in terms of the size of the dwelling.

Discrimination on the housing market can limit the opportunities of ethnic minorities to improve their housing situation (South and Crowder 1998). Also in the Netherlands, lending institutions are found to have less trust in ethnic minorities, who therefore are less likely to get a (high) mortgage than natives (Aalbers 2007, 2013) and landlords in the private rental sector might prefer native renters over ethnic minorities (Kullberg et al. 2009). Within the social housing sector in the Netherlands, there are less opportunities for open discrimination (Kullberg 2002); however, a lower Dutch language proficiency or lower understanding of the housing allocation system can reduce the opportunities of ethnic minorities to realise their moving desires (Bolt 2001; Van Kempen and Idamir 2003). Also fear of discrimination can limit the moving opportunities of ethnic minorities as it might prevent them from moving to majority concentration neighbourhoods (Hanhoerster 2013; Phillips et al. 2007). Research in the Netherlands shows that many ethnic minorities fear that they will not be accepted or will be unable to get in touch with their neighbours in majority concentration neighbourhoods and therefore choose not to move to these neighbourhoods (Kullberg et al. 2009).

Furthermore, social ties within the neighbourhood may prevent residential mobility (Dawkins 2006; Parkes et al. 2002). A social network within the neighbourhood can provide cheap alternatives to costly services such as day care for children, transportation and recreation (Connerly 1986; DaVanzo 1981). This type of social capital is location specific and difficult to redevelop after moving (DaVanzo 1981). Especially low-income households and ethnic minorities are found to rely on this type of social capital (Portes 1998). These groups thus have higher (social) costs of leaving the neighbourhood and will therefore be less likely to leave. Possibly, they are also less successful in leaving their neighbourhood even if they do have a desire to leave. Finally, local housing market opportunities and the macro-level economic situation affect opportunities of individuals to find a better housing situation and thus to realise their desire to move (De Groot et al. 2011; Lu 1998).

Many studies test whether individuals actually *realise* their desire to move. These studies often find a large discrepancy between desires, expectations or intentions to move^1^ and actual moving behaviour (Crowder 2001; De Groot et al. 2011; Kan 1999; Landale and Guest 1985; Lee et al. 1994; Lu 1999; Moore 1986). The majority of people who stated they want to move do not realise their moving desire within one or two years (Crowder 2001; De Groot et al. 2011; Kan 1999; Lu 1999). High-income households are found to be more likely to realise their desires (Boschman and De Groot 2011; Crowder 2001; Moore 1986). Blacks or ethnic minorities are found to be less successful in realising their desire to move (Boschman and De Groot 2011; Crowder 2001; De Groot et al. 2011; Kan 1999; Moore 1986). The same often applies to larger households (De Groot et al. 2008; Kan 1999). For some characteristics, findings are mixed. Older people are found to be less likely to realise their desire to move (De Groot et al. 2008; Moore 1986), but Kan (1999) finds no significant effect of age. Owners are found to be more successful by some researchers (De Groot et al. 2008) and less successful by others (Kan 1999; Moore 1986).

### Linking segregation and residential mobility: the role of the neighbourhood

According to residential mobility theory, households reveal a desire to move if they are dissatisfied with their current housing situation (Brown and Moore 1970; Wolpert 1965). In the households’ evaluation of their housing situation, both dwelling and neighbourhood characteristics are important (Clark et al. 2006). Neighbourhood change can create a discrepancy between the preferred and the actual housing situation and therefore trigger a desire to move (Wolpert 1965). Moreover, impending or planned events in life course trajectories, such as changes in household composition (starting a family) or socioeconomic situation (income increase), will result in a changing evaluation of both the dwelling and the neighbourhood (Lee et al. 1994). A neighbourhood that was in line with the residential preferences of a couple might not meet their needs and standards anymore once they are planning to start a family. Hence, neighbourhood characteristics such as low school quality or nuisance, which were not considered problematic previously, can suddenly fuel a desire to leave the neighbourhood.

Much research has been done on which neighbourhood characteristics are a reason to want to leave the neighbourhood, especially on the role of the ethnic or racial composition of the neighbourhood. In the USA, Schelling (1971) hypothesises that individuals do not want to be a minority in their neighbourhood and thus move out if the share of ‘others’ is higher than the share of their own group. Farley et al. (1978) confronted White individuals with hypothetical neighbourhoods with various shares of Black households and no information on other neighbourhood characteristics. Following Farley et al. (1978), various researchers have shown that increasing shares of Whites describe the neighbourhood as undesirable or state they would try to move out, if the share of Black households increases (Farley et al. 1978; Krysan 2002; Krysan et al. 2009).

Both researchers in the USA and Europe have tested the effect of various neighbourhood characteristics on the desire to leave the neighbourhood (Van Ham and Feijten 2008), neighbourhood outflow (Ellen 2000; Van Ham and Clark 2009), neighbourhood satisfaction (Dekker 2013; Harris 2001; Swaroop and Krysan 2011) or dwelling prices (Harris 1999). They find that in neighbourhoods with higher shares of ethnic or racial minorities, more people (want to) leave the neighbourhood and neighbourhood satisfaction is lower. However, critics claim that this is not necessarily caused by the ethnic composition of the neighbourhood (Ellen 2000; Harris 2001). Other neighbourhood characteristics, such as dwelling prices or poverty, might be more important in the evaluation of the neighbourhood and might be correlated with the ethnic composition (Havekes et al. 2016).

The effect of the neighbourhood ethnic or racial composition on moving desires or outward mobility is less strong for ethnic or racial minorities than for the native majority (Pais et al. 2009; Van Ham and Clark 2009; Van Ham and Feijten 2008). Black households are found to have a preference for mixed neighbourhoods and to be more tolerant than Whites to neighbourhoods with different racial compositions (Farley et al. 1978; Krysan et al. 2009). Also in the Netherlands, especially the native majority is found to (want to) leave minority concentration neighbourhoods (Bolt and Van Kempen 2010; Van Ham and Feijten 2008).

Apart from neighbourhood ethnic or racial composition, other neighbourhood characteristics may be related to neighbourhood satisfaction or (desired) mobility out of the neighbourhood. Harris (2001) finds a negative effect on neighbourhood satisfaction of poverty, crime, deterioration and bad schools. Dekker (2013) finds lower neighbourhood satisfaction in neighbourhoods with low incomes and low dwelling values. However, Ellen (2000) and Van Ham and Clark (2009) find no significant effect of neighbourhood income on mobility out of the neighbourhood. Possibly, households in poverty neighbourhoods are less satisfied and more often want to leave the neighbourhood, but do not succeed in realising their desire to leave.

## Ethnic minority groups in the Netherlands

The four largest minority groups in the Netherlands are Turks (2.4 %), Moroccans (2.2 %), Surinamese (2.1 %) and Antilleans (0.9 %). Besides these four groups, we include other non-Western minorities (4.3 %) and Western minorities (9.5 %) (percentages over 2014, source: Statistics Netherlands (2016)). We use the Statistics Netherlands definition of ethnic groups. Non-Western minorities are people of whom at least one parent is born in Africa, Latin America or Asia (except Indonesia and Japan). Western minorities are people of whom at least one parent is born in another country outside the Netherlands. The immigration of Turks and Moroccans started in the 1960 when they were recruited as guest workers. Especially unskilled labourers from the poorest rural areas were recruited, to solve the shortages of low-paid unskilled workers on the labour (Castles 2006). In the 1970s and 1980s, the immigrant population increased further because of family reunification and family formation. This migration history explains the in general low educational level of Turks and Moroccans in the Netherlands.

Surinamese and Antilleans are immigrants from former Dutch colonies. Most Surinamese came to the Netherlands after the declaration of independence of Surinam in 1975. Until the 1990s, Antilleans came mainly to the Netherlands to acquire higher education. More recently, more underprivileged Antilleans came to the Netherlands to find a job. Surinamese and Antilleans in the Netherlands have a higher Dutch language proficiency because of the colonial history, are higher educated and more often have a job and a high income than Turks and Moroccans (Dagevos 2007). Ethnic residential segregation in cities in the Netherlands is moderate to low compared with other European countries and higher for Turks and Moroccans than for Surinamese and Antilleans (Musterd and Ostendorf 2009). While Turks, Moroccans and Surinamese generally have been in the Netherlands for a long time, among Antilleans and especially among the category of other non-Western minorities there are also many more recent immigrants. Because of their short duration of stay in the Netherlands, these groups might not have established a good position on the housing market yet and therefore might more often (want to) move (Åslund 2005; Bolt 2001; Boschman and Van Dam 2012). Antilleans are known to live in the worst quality housing (Kullberg et al. 2009) and therefore to more often (want to) move (Boschman and De Groot 2011). Western minorities are most comparable to the native majority in their socio-economic status and their position on the housing market.

## Hypotheses

Non-Western ethnic minorities have been found to be less successful in realising their desires to move. In addition, they rely more on informal social capital and will therefore have higher social costs of leaving the neighbourhood (Portes 1998). Our first hypothesis therefore is that *non*-*Western ethnic minorities are less successful than native Dutch people in realising their wish to leave the neighbourhood* (Hypothesis 1).

Secondly, we will test which groups are successful in leaving which neighbourhoods. Ethnic minorities are found to leave minority concentration neighbourhoods less often than native residents. Hence, we hypothesise that *ethnic minorities are less successful than native Dutch people in leaving minority concentration neighbourhoods, even if they expressed a desire to do so* (Hypothesis 2). Discrimination on the housing market or the strength of networks might prevent ethnic minorities to leave minority concentration neighbourhoods. However, for the same reasons, they might also be less likely to have a desire to leave these neighbourhoods and might be equally successful if they do have a desire to leave.

## Data, selections and methods

For our study, we use a combination of survey data and register data. We use data from the Housing Research Netherlands survey (WoON 2006 and WoON 2009^2^), a housing survey that is representative for the Dutch population aged 18 years and older (not living in institutions). We combine these data with longitudinal register data on residential mobility histories of the complete population of the Netherlands (SSD). Therefore, we can follow the survey respondents over time and test whether they leave their neighbourhood in the two years following the survey and which neighbourhoods they move to. We enriched this data set with data from Statistics Netherlands on neighbourhood characteristics such as the share of rented dwellings, the average neighbourhood income and the share of various ethnic groups.

We used administrative neighbourhoods (*buurten*) as defined by Statistics Netherlands. Within urban areas, neighbourhoods are small, with an average size of 1.4 km^2^ and an average number of 6000 inhabitants. They often have natural borders. These neighbourhoods are the lowest administrative area level in the Netherlands. Therefore, more people will be found successful in leaving their neighbourhood than with other, larger definitions of neighbourhoods, such as postal code areas or districts. By choosing the smallest possible neighbourhood definition, we minimise the number of people who successfully left their perceived neighbourhood, but who in our data appear as movers within the neighbourhood.

In the Housing Research Netherlands survey, respondents are asked about their personal characteristics, household situation, housing situation and moving wishes. On a five-point Likert scale, respondents are asked to agree or disagree with: ‘If possible, I would leave the neighbourhood’. In total, there are 142,073 respondents, 64,005 in the 2006 housing survey and 78,068 in the 2009 survey. For respondents who are included in both surveys (870 respondents), we randomly selected only one survey year to ensure independence of observations. 3298 respondents (2 %) in the survey could not be traced in the register data 2 years after the interview, probably because they died or emigrated, and were therefore excluded from the data. Also (adult) children living at the parental home, respondents residing in another households dwelling, respondents who were planning to move and already found a new dwelling and respondents with missing data on neighbourhood characteristics (17,287 respondents) were excluded, which leaves 120,618 respondents in our sample.

Segregation research has traditionally focused on urban areas, while residential mobility research focuses on both urban and rural areas (Boschman 2015). Especially in urban areas, neighbourhoods differ in ethnic or income composition and neighbourhood characteristics can affect mobility desires and behaviour. This paper studies selective mobility with the aim to better understand segregation and therefore only focuses on urban areas. In the Netherlands, there are very large differences between urban regions in the share of ethnic minorities. In the four largest cities, the share of ethnic minorities is much higher than in other urban areas, which would make the results incomparable. To be able to study effects of the ethnic composition, we thus only selected the urban regions of the four largest cities. We included 39,549 respondents of which 6836 (17 %) state they (totally) agree with the statement ‘if possible I would leave the neighbourhood’ (Table 1).

**Table 1:** Leaving the neighbourhood, wishes and behaviour, percentages per ethnic group (*N* = 39,549). *Source*: Own calculations based on WoON 2006 and 2009 and SSB, provided by Statistics Netherlands

	Wants to leave	Leaves	Leaves (within wants to leave	Leaves (within does not want to leave)
Native Dutch	15.1	9.9	24.8	7.2
Moroccans	30.0	12.6	20.9	9.0
Turks	27.4	10.7	16.1	8.7
Antilleans	26.7	17.5	34.5	11.3
Surinamese	24.8	10.7	20.9	7.4
Other non-Western minorities	28.4	15.7	22.7	12.9
Total non-Western	27.2	12.2	21.8	9.6
Western minorities	17.7	10.2	26.2	6.8
Total	17.3	10.4	24.2	7.5

Below we focus on the 6836 respondents who stated that they want to leave their neighbourhood. We estimated a binary logistic regression model of who is successful in realising their wish to leave the neighbourhood. In this model, we included both personal characteristics and neighbourhood characteristics. Because we included variables on both neighbourhood and individual level, we used clustered standards errors on neighbourhood level.^3^ On the individual level, we include ethnicity, age, household type, educational level and income. In addition to our key variable ‘if possible I would leave the neighbourhood’, respondents are also asked whether they have a desire to move or expect to be forced to move within 2 years. Forced moves are commonly caused by demolition, in the context of urban restructuring (Kleinhans 2012). Based on these questions, we created an extra indicator of mobility desires and expectations. We control for this variable and for tenure, dwelling type, crowding^4^ and dwelling satisfaction^5^ as these variables have been found to be highly related to mobility behaviour (Coulter 2013; De Groot et al. 2011). On the neighbourhood level, we control for average dwelling values, the share of rented dwellings, average income and the share of non-Western minorities to test whether there are differences between neighbourhood types in residents realising their moving desires. We also include controls for the four urban regions and for urban density at municipal level^6^ as the housing market situation might differ between the urban regions and between high and low urban density municipalities. Finally, we include interaction effects of neighbourhood income and neighbourhood share of non-Western minorities with personal characteristics to test which groups are successful in leaving which neighbourhoods (Table 2).

**Table 2:** Descriptive statistics of the independent variables*. *Source*: Own calculations based on WoON 2006 and 2009 and SSB, provided by Statistics Netherlands

	Mean	SE
*Personal characteristics*		
Ethnicity		
Native Dutch	0.65	
Moroccan	0.04	
Turkish	0.05	
Surinamese	0.07	
Antillean	0.02	
Western minority	0.09	
Other non-Western	0.07	
Year 2009	0.58	
Moving wish		
Wish to move	0.71	
Expect forced move	0.01	
No moving wish	0.28	
Age		
18–24	0.08	
25–34	0.24	
35–44	0.23	
45–54	0.19	
55–64	0.14	
65–74	0.08	
75+	0.05	
Household type		
Single	0.34	
Couple	0.25	
Family with children	0.26	
Single parent	0.11	
Non-family household	0.03	
Education level		
Low	0.38	
Middle	0.34	
High	0.28	
Income (standardised)	0.00	1.00
Owner	0.34	
Satisfied with dwelling	0.64	
Dwelling type (ref = single family dwelling)	0.66	
Single family dwelling	0.30	
Apartment	0.66	
Other housing unit	0.04	
Overcrowded	0.24	
Undercrowded	0.28	
*Neighbourhood characteristics*		
Average dwelling value neighbourhood (1000×)	177.54	71.71
Share of rented dwellings neighbourhood	61.28	21.65
Average-income neighbourhood (standardised)	0.00	1.00
% Non-Western minorities (standardised)	0.00	1.00
Urban density		
Very high	0.62	
High	0.26	
Average	0.08	
Low	0.03	
Very low	0.01	
Urban region		
Amsterdam	0.24	
Utrecht	0.10	
Rotterdam	0.29	
The Hague	0.37	

* Statistics Netherlands does not allow disclosure of minimum and maximum values

## Results

### Ethnic differences in leaving wishes and behaviour

In total, there are 39,549 inhabitants of the four urban regions of which 6836 (17 %) (totally) agreed with the statement ‘if possible I would leave the neighbourhood’ (see Table 1). Most respondents with a desire to leave their neighbourhood do not realise this desire within 2 years. Only 24 % of the respondents with a desire to leave have left their neighbourhood within 2 years and 7.5 % of the respondents *without* a desire to leave have left their neighbourhood in the 2 years after the survey.

Non-Western minorities more often want to leave their neighbourhood than native Dutch respondents and Western minorities. Turkish, Moroccan, Surinamese and other non-Western minorities less often succeed in leaving their neighbourhood than Western minorities and native Dutch respondents with a wish to leave their neighbourhood. Antilleans, however, more often than native Dutch respondents, realise their wish to leave the neighbourhood. Non-Western minorities, especially Antilleans and the category of other non-Western minorities, are most likely to leave their neighbourhood when they did not have a desire to leave (see Table 1).

These ethnic differences in moving wishes and behaviour might be (partly) explained by ethnic differences in socio-economic, housing and neighbourhood situation. Ethnic groups differ in average income, age, tenure and neighbourhood ethnic composition, and all these variables are known to affect moving wishes and behaviour. To test whether ethnicity has a separate effect on the realisation of wishes to leave the neighbourhood, we estimate multivariate models in which we take into account all sorts of personal and neighbourhood characteristics.

### Who realise their desire to leave the neighbourhood?

In Hypothesis 1, we stated that non-Western ethnic minorities are less successful in realising their desire to leave the neighbourhood. Models 1 and 2 (see Table 3) are logistic regression models that estimate which personal and neighbourhood characteristics are related to realising a desire to leave. These models are estimated on the 6836 respondents who state they want to leave their neighbourhood. We control for survey year, for whether people have a desire to move or expect to be forced to move, for personal characteristics such as age, household type, income and dwelling characteristics and for neighbourhood characteristics.

**Table 3:** Logistic regression models: realising a wish to leave the neighbourhood (*N* = 6836). *Source*: Own calculations based on WoON 2006 and 2009 and SSB, provided by Statistics Netherlands

	Model 1	Model 2
	OR (SE)	OR (SE)
*Personal characteristics*		
Ethnicity (ref = native Dutch)		
Moroccans	0.517 (0.098)**	0.607 (0.128)*
Turks	0.416 (0.074)**	0.479 (0.118)**
Surinamese	0.741 (0.097)*	0.745 (0.111)*
Antilleans	1.066 (0.193)	1.066 (0.206)
Western minorities	1.006 (0.116)	0.997 (0.116)
Other non-Western minorities	0.642 (0.083)**	0.662 (0.09)**
Year 2009	0.781 (0.061)**	0.788 (0.061)**
Moving wish (ref = wish)		
Expect forced move	1.305 (0.439)	1.290 (0.435)
No moving wish	0.271 (0.024)**	0.271 (0.024)**
Age (18–24 = ref)		
25–34	0.689 (0.077)**	0.699 (0.079)**
35–44	0.399 (0.05)**	0.404 (0.052)**
45–54	0.282 (0.039)**	0.288 (0.04)**
55–64	0.263 (0.036)**	0.268 (0.037)**
65–74	0.277 (0.046)**	0.282 (0.047)**
75+	0.461 (0.084)**	0.471 (0.085)**
Household type (ref = single)		
Couple	1.201 (0.120)	1.215 (0.123)
Family with children	0.833 (0.111)	0.845 (0.114)
Single parent	0.667 (0.089)**	0.675 (0.090)**
Non-family household	1.637 (0.256)**	1.651 (0.262)**
Education level (ref = low)		
Middle	0.969 (0.078)	0.971 (0.078)
High	1.028 (0.089)	1.027 (0.090)
Income (standardised)	1.129 (0.043)**	1.111 (0.047)**
Owner	0.701 (0.066)**	0.704 (0.066)**
Satisfied with dwelling	0.883 (0.057)	0.881 (0.057)*
Dwelling type (ref = single family dwelling)		
Apartment	1.266 (0.115)*	1.267 (0.116)*
Other housing unit	2.006 (0.376)**	2.006 (0.376)**
Overcrowded	1.203 (0.101)*	1.214 (0.102)*
Undercrowded	0.96 (0.092)	0.963 (0.093)
*Neighbourhood characteristics*		
Average dwelling value neighbourhood	1.000 (0.001)	1.000 (0.001)
Share of rented dwellings neighbourhood	0.998 (0.002)	0.998 (0.002)
Average-income neighbourhood (standardised)	1.015 (0.065)	1.018 (0.065)
% Non-Western minorities (standardised)	1.103 (0.057)	1.114 (0.064)
Urban density (ref = very high)		
High	1.174 (0.140)	1.179 (0.139)
Average	0.993 (0.144)	0.992 (0.143)
Low	0.877 (0.193)	0.889 (0.194)
Very low	0.822 (0.293)	0.823 (0.294)
Utrecht urban region	1.001 (0.120)	1.007 (0.121)
Rotterdam urban region	1.190 (0.129)	1.189 (0.128)
The Hague urban region	1.165 (0.100)	1.175 (0.101)
*Interactions*		
Moroccan * share of non-Western minorities		0.807 (0.150)
Turkish * share of non-Western minorities		0.850 (0.148)
Surinamese * share of non-Western minorities		0.976 (0.119)
Antillean * share of non-Western minorities		0.979 (0.182)
Western * share of non-Western minorities		1.196 (0.143)
Other non-Western * share of non-Western minorities		0.891 (0.112)
Income * average-income neighbourhood		1.031 (0.026)
Intercept	1.125 (0.334)	1.119 (0.325)
*R* ^2^	0.115	0.116

* *p* < 0.05; ** *p* < 0.01

When these characteristics are taken into account, we find that Turks (OR 0.416, *p* < 0.01), Moroccans (OR 0.517, *p* < 0.01), Surinamese (OR 0.741, *p* = 0.022) and the category of other non-Western minorities (OR 0.642, *p* < 0.01) are less successful than native Dutch in leaving their neighbourhood. Antilleans (OR 1.066, *p* = 0.724) and Western minorities (OR 0.1006, *p* = 0.961) are equally successful as native Dutch respondents. People are less successful in realising desires to leave their neighbourhood in 2009 than in 2006 (OR 0.781, *p* < 0.01). The economic crisis, including increasing financial insecurity, decreasing dwelling prices and lower levels of new housing construction, has led to lower residential mobility (Priemus 2010), also among people who want to leave their neighbourhood. On neighbourhood level, we included average dwelling value, share of rented dwellings, average income, share of non-Western minorities and urban density, as well as dummy variables that measure the differences between the four urban regions. However, none of these variables has significant effect on the realisation of desires to leave the neighbourhood. Neighbourhood characteristics thus have no effect on the *realisation* of desires to leave the neighbourhood. Earlier research has found an effect of neighbourhood characteristics on the *desire* to leave the neighbourhood (Van Ham and Feijten 2008; Lee et al. 1994) and on mobility out of the neighbourhood (Bolt and Van Kempen 2003; South and Crowder 1998; Van Ham and Clark 2009). However, we find that neighbourhood characteristics do not affect mobility out of the neighbourhood *conditional on desires to leave*. That is, when taken into account that people *want to leave* their neighbourhood, neighbourhood characteristics no longer have an effect on whether people leave their neighbourhood.

Hypothesis 1 states that non-Western minorities are less successful in realising a desire to leave their neighbourhood. In model 1, we find that Turks, Moroccans, Surinamese and other non-Western minorities are less successful than native Dutch in realising their desire to leave their neighbourhood. For these groups, we can thus confirm Hypothesis 1. However, Antilleans are equally successful as native Dutch in realising their desire to leave their neighbourhood. Antilleans live in the worst housing conditions (Kullberg et al. 2009) and most often move, also if they have no desire to move (Boschman and De Groot 2011). This might explain why they realise desires to leave the neighbourhood more often than other non-Western minority groups.

In model 2, cross-level interactions between neighbourhood income and personal income and between the share of ethnic minorities in the neighbourhood and ethnicity on individual level are included. We use this model to test Hypothesis 2, which states that ethnic minorities are *especially* less successful in leaving minority concentration neighbourhoods, even if they express a desire to do so. Although the interaction effects go in the hypothesised direction, they are not significant. For none of the ethnic groups, the share of non-Western minorities has significant effect on their realisation of desires to move. Thus, although Moroccans, Turks, Surinamese and other non-Western minorities are less successful in realising desires to leave their neighbourhood, they are not *especially* unsuccessful in leaving minority concentration neighbourhoods. Also the effect of average income in the neighbourhood on realising desires to move does not depend on *personal* income; although households with lower incomes are less successful in realising desires to leave their neighbourhood, they are not *especially* unsuccessful in leaving low-income neighbourhoods. Based on these outcomes, Hypothesis 2 can be rejected (Table 3).

## Conclusions and discussion

A substantial body of the literature has analysed the characteristics of people who want to leave their neighbourhood and who actually do leave their neighbourhood. Earlier research has shown that ethnic minorities are less likely than natives to leave ethnic minority concentration neighbourhoods. This mechanism can lead to (re)production of segregation. However, we do not know whether this can be explained by ethnic minorities being less likely than natives to *want to* leave these neighbourhoods—which would indicate that segregation is voluntary—or whether this is explained by ethnic minorities being less successful in realising desire to leave these neighbourhoods.

Several studies have shown that the native majority is more likely than ethnic minorities to want to leave neighbourhoods with higher shares of ethnic minorities (Lee et al. 1994; Van Ham and Feijten 2008). Other studies, however, reveal that most people with a desire to move do not realise this desire and that especially ethnic minorities are unsuccessful in realising moving desires (Crowder 2001; De Groot et al. 2011). Both differences in desires and differences in *realisation of desires* can therefore lead to selective mobility patterns.

This paper has focused on individuals with a *desire to* *leave their neighbourhood*. We investigated ethnic differences in the extent to which people are able to fulfil this desire. In line with our hypothesis, we find that Turks, Moroccans, Surinamese and other non-Western ethnic minorities are less successful than native Dutch in realising a desire to leave their neighbourhood. Antilleans and Western minorities are, however, not significantly less successful than natives. Non-Western minorities (except Antilleans) and low-income households who want to leave their neighbourhood are less likely than natives to leave and thus more likely to be ‘trapped’ in undesired neighbourhoods.

Several studies have found that neighbourhood conditions affect the desire to leave the neighbourhood (Lee et al. 1994; Van Ham and Feijten 2008) and actual mobility out of the neighbourhood (Bolt and Van Kempen 2003; Ellen 2000; Musterd et al. 2014; Van Ham and Clark 2009). However, we have found no effect of neighbourhood characteristics on the realisation of wishes to leave the neighbourhood. Although neighbourhood characteristics have been found to affect both desires to leave and mobility out of the neighbourhood, they do not affect mobility out of the neighbourhood *conditional on the desire to leave*. For individuals who want to leave, neighbourhood characteristics such as the share of minorities or average income do not affect their probability of success.

Many studies show that ethnic minorities less often than natives leave ethnic minority concentration neighbourhoods. We studied whether ethnic minorities are also less successful in leaving ethnic minority concentration neighbourhoods *if they have expressed a desire to leave their neighbourhood*. For none of the ethnic groups, the share of ethnic minorities in the neighbourhood has a significant effect on their realisation of desires to leave. Non-Western minorities are equally (un)successful if they want to leave ethnic minority concentration neighbourhoods as if they want to leave neighbourhoods with lower shares of minorities. The finding in earlier research that ethnic minorities are less likely to leave ethnic minority concentration neighbourhoods is thus most likely explained by the fact they are less likely to *want to leave* these neighbourhoods.

This paper provides new insights in selective mobility because it shows selectivity in the discrepancy between desires to leave and actual mobility out of the neighbourhood. Regardless of neighbourhood characteristics, Moroccans, Turks, Surinamese and other non-Western minorities are found to be less successful in realising desires to leave. Since these groups often live in ethnic minority concentration neighbourhoods, their inability to realise their desire to leave can keep segregation at relatively high levels.

## Footnotes

1 In residential mobility literature, many studies have been done on the realisation of mobility desires, intentions or expectations. Most papers do not pay attention to the differences between these concepts; however, Coulter et al. (2011) show that desires and expectations are different and have a different impact on subsequent behaviour. In our research, we use the terms desires or wishes, because in our data, people are asked about their moving desires and their desires to leave the neighbourhood. We are, however, aware that other researchers have used other concepts which make their outcomes less comparable.

2 We combine data from two survey years to increase the number of respondents in our analyses.

3 For the respondents in WoON 2009, we used neighbourhood characteristics from 2009, for the respondents from WoON 2006 we used neighbourhood characteristics from 2006. A neighbourhood in 2009 thus has different neighbourhood characteristics than the same neighbourhood in 2006 and therefore is considered to be a different neighbourhood. The 6836 respondents are distributed over 1416 unique neighbourhoods.

4 Crowding is calculated based on the number of rooms per person where ≤1 room per person is overcrowded and ≥2 rooms per person is undercrowded.

5 This variable is based on the question ‘How satisfied are you with your dwelling?’ People who indicated that they were (very) satisfied are coded as 1, while people who are ‘not satisfied/not dissatisfied’ or (very) dissatisfied are coded 0.

6 Urban density is an often used municipality level indicator of urbanisation created by Statistics Netherlands and based on the number of addresses per km^2^ (see further CBS.nl).
